# Evolutionary significance of the variation in acoustic communication of a cryptic nocturnal primate radiation (*Microcebus* spp.)

**DOI:** 10.1002/ece3.6177

**Published:** 2020-03-12

**Authors:** Alida Frankline Hasiniaina, Ute Radespiel, Sharon E. Kessler, Mamy Rina Evasoa, Solofonirina Rasoloharijaona, Blanchard Randrianambinina, Elke Zimmermann, Sabine Schmidt, Marina Scheumann

**Affiliations:** ^1^ Institute of Zoology University of Veterinary Medicine Hannover Hannover Germany; ^2^ Department of Psychology Faculty of Natural Sciences University of Stirling Stirling Scotland; ^3^ Department of Anthropology Durham University Durham UK; ^4^ Faculty of Science, Technology and Environment University of Mahajanga Mahajanga Madagascar

**Keywords:** acoustic communication, evolution, genetic drift, mouse lemur, primate, selection

## Abstract

Acoustic phenotypic variation is of major importance for speciation and the evolution of species diversity. Whereas selective and stochastic forces shaping the acoustic divergence of signaling systems are well studied in insects, frogs, and birds, knowledge on the processes driving acoustic phenotypic evolution in mammals is limited. We quantified the acoustic variation of a call type exchanged during agonistic encounters across eight distinct species of the smallest‐bodied nocturnal primate radiation, the Malagasy mouse lemurs. The species live in two different habitats (dry forest vs. humid forest), differ in geographic distance to each other, and belong to four distinct phylogenetic clades within the genus. Genetically defined species were discriminated reliably on the phenotypic level based on their acoustic distinctiveness in a discriminant function analysis. Acoustic variation was explained by genetic distance, whereas differences in morphology, forest type, or geographic distance had no effect. The strong impact of genetics was supported by a correlation between acoustic and genetic distance and the high agreement in branching pattern between the acoustic and molecular phylogenetic trees. In sum, stochastic factors such as genetic drift best explained acoustic diversification in a social communication call of mouse lemurs.

## Graphical abstract

The study shows that eight cryptic mouse lemur species differ in the acoustic parameters of their agonistic Tsak calls. This acoustic diversification can be best explained by stochastic factors such as genetic drift rather than by ecology.

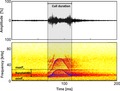

## 1 INTRODUCTION

Phenotypic diversity is the substrate for speciation and the evolution of species diversity. Variation in signaling systems may mediate discrimination within and between species (Wilkins, Seddon, & Safran, 2013; Zimmermann, 2016). Current comparative bioacoustic research suggests that three major selective forces drive acoustic variation within and among populations favoring speciation and evolution (Wilkins et al., 2013): ecological selection, sexual selection, and genetic drift. Ecological selection refers to a genetic adaptation to a particular environment (Wilkins et al., 2013). For example, in Darwin finches, climate constraints shape feeding ecology and therefore bill shape, which affects the structure of trill calls in their mating song (Podos, 2010). Thus, the ecological selection for beak size affects acoustic divergence between different Darwin finch morphs reinforced by assortative mating. Further, acoustic adaptation to sound transmission characteristics of the environment, or ambient noise, has been shown to affect the structure of vocalizations (acoustic adaptation hypothesis; Brown & Waser, 2017) in insects, birds, anurans, and mammals (McNett & Cocroft, 2008; for review, see Boncoraglio & Saino, 2007, Ey & Fischer, 2009). For example, bird vocalizations have a lower maximum frequency in closed versus open habitats (Boncoraglio & Saino, 2007; Ey & Fischer, 2009). However, Ey and Fischer (2009) did not find general rules for environment‐related acoustic variations of calls in anurans and mammals, suggesting that environmental adaptations may be constrained by other call‐related factors such as their behavioral context. Sexual selection results from competition for mating partners (Wilkins et al., 2013). Irwin, Thimgan, and Irwin (2008) found differences in the pattern of geographic variation between calls and songs in greenish warblers, which might be explained by sexual selection on the songs used for mating. In contrast to the adaptive mechanisms, genetic drift is a stochastic process reflecting random changes in the frequencies of gene variants (alleles) within a population (Wilkins et al., 2013). Due to the fact that in some studies, genetic distance correlates strongly with geographic distance (e.g., Campbell et al., 2010; Irwin et al., 2008; Pröhl, Hagemann, Karsch, & Hobel, 2007; Thinh, Hallam, Roos, & Hammerschmidt, 2011), geographic distance has often been used as a proxy for genetic distance.

In mammals, various studies address micro‐ and macrogeographic acoustic variation in communication calls across populations, or closely related species (e.g., Macroscelidea: Faurie, 1996; Cetacea: Baron, Martinez, Garrison, & Keith, 2008, Samarra, Deecke, Simonis, & Miller, 2015; Artiodactyla: Gebler & Frey, 2005, Volodin, Nahlik, Tari, Frey, & Volodina, 2019; Carnivora: Perry & Terhune, 1999, Page, Goldsworthy, Hindell, & Mckenzie, 2002, Mizuguchi, Mitani, & Kohshima, 2016; Rodentia: Ancillotto et al., 2017, Chen, Su, Qin, & Liu, 2017; Chiroptera: Schöner, Schöner, & Kerth, 2010, Schuchmann & Siemers, 2010; Scandentia: Esser, Schehka, & Zimmermann, 2008; and Primates: Méndez‐Cárdenas, Randrianambinina, Rabesandratana, Rasoloharijaona, & Zimmermann, 2008, Fischer & Hammerschmidt, 2020). Acoustic variation across primate species has been related to selective forces (e.g., Braune, Schmidt, & Zimmermann, 2008; Masters, 1991; Schneider, Hodges, Fischer, & Hammerschmidt, 2008) or stochastic processes (e.g., Adret et al., 2018; Méndez‐Cárdenas et al., 2008; Meyer et al., 2012; Thinh et al., 2011). In singing mice, both selective forces and stochastic processes were studied revealing genetic drift as a major driving force for acoustic divergence (Campbell et al., 2010). To evaluate the effects of these two factors in primates, we studied the mouse lemur radiation.

Mouse lemurs, endemic to the island of Madagascar, provide a unique primate radiation for exploring the significance of vocal communication for species diversity and evolution in mammals. Mouse lemurs are described as a cryptic, species‐rich taxon (Hotaling et al., 2016; Yoder et al., 2000) since species display rather small differences in body size and mass (30–80 g) and other obvious phenotypic traits. During the last 25 years, field studies associated with intensive sampling efforts for genetic analyses and technological advances in molecular genetics and phylogenetic research led to the description of currently 24 different species (e.g., Andriantompohavana et al., 2006; Hotaling et al., 2016; Louis et al., 2006; Louis et al., 2008; Olivieri et al., 2007; Radespiel et al., 2012; Rasoloarison, Weisrock, Yoder, Rakotondravony, & Kappeler, 2013; Rasolooarison, Goodman, & Ganzhorn, 2000; Zimmermann, Cepok, Rakotoarison, Zietemann, & Radespiel, 1998). Species delimitation was so far mainly based on mtDNA divergence, some morphological comparisons, and allopatric distribution patterns, and made use of the phylogenetic species concept (Radespiel et al., 2008, 2012; Rasoloarison et al., 2013; Schneider et al., 2008; Zimmermann & Radespiel, 2014). Most of the genetically defined species are threatened by fragmentation of their habitats or natural habitat loss and thus classified in the IUCN Red List as endangered or even critically endangered (Schwitzer et al., 2014). At present, the species diversity within this genus is controversial, with some taxonomists (Isaac, Mallet, & Mace, 2004; Markolf, Brameier, & Kappeler, 2011; Tattersall, 2013; Zachos et al., 2013), suggesting that it may reflect “taxonomic inflation.”

Most of the described species show local to regional endemism with distributions in either dry deciduous, or rain, forest types across Madagascar, where species most often limited to a single so‐called “inter‐river system” (IRS, Olivieri et al., 2007). In contrast, the gray mouse lemur (*Microcebus murinus*) shows a broad distribution range across dry deciduous forests from the northwest to the southeast encompassing several IRSs, often resulting in sympatry with other mouse lemur species. *M. murinus* most likely expanded very recently into the regions of sympatry (Schneider, Chikhi, Currat, & Radespiel, 2010; Yoder et al., 2000). Survival of the nocturnal mouse lemurs in their dense three‐dimensional forest environment is strongly linked to olfaction and audition (Bunkus, Scheumann, & Zimmermann, 2005; Hohenbrink, Mundy, Zimmermann, & Radespiel, 2013; Hohenbrink, Radespiel, & Mundy, 2012; Kappel, Hohenbrink, & Radespiel, 2011; Rahlfs & Fichtel, 2010), since vision is environmentally and physiologically constrained in the dark (Charles‐Dominique & Petter, 1980; Piep, Radespiel, Zimmermann, Schmidt, & Siemers, 2008; Valenta et al., 2013). Mouse lemurs evolved a set of acoustically complex vocalizations in the audible and/or ultrasonic range conveying indexical and emotional information, and governing agonistic conflicts, matings, mother–infant, or group, reunions, or antipredator strategies (Fichtel, 2016; Scheumann, Linn, & Zimmermann, 2017; Zimmermann, 2010, 2018).

Based on the high cryptic species diversity and the important role of vocalizations for social communication, mouse lemurs provide an excellent primate model group to explore current hypotheses for acoustic divergence driving speciation and evolution in a closely related radiation of mammals. We quantify acoustic variation of a common call type in eight species of mouse lemurs originating from seven geographically distinct regions in northwestern, northern, and eastern Madagascar. These species belong to four phylogenetic clades and live in different forest types. As species‐specific calls are a prerequisite to investigate the impact of selective and stochastic forces on vocal behavior in mouse lemurs, we first tested the hypothesis that the calls of the eight species differ in their acoustic characteristics. Second, we evaluated whether these species‐specific differences can be explained by morphological differences between the species such as body size and vocal tract length (e.g., Ey, Pfefferle, & Fischer, 2007; Masters, 1991; Plotsky, Rendall, Riede, & Chase, 2013). We predict that if morphometry explains species‐specific differences, acoustic data correlate with morphometric data related to the body and head size. Third, we tested whether ecology drives acoustic divergence to optimize transmission using forest type as a proxy of ecology. According to the literature in mammals, we predicted that species living in humid forest (closed habitats) have calls with a longer duration, more narrow band, and lower fundamental frequency than species living in dry forest (more open habitats; e.g., Brown & Waser, 2017; Ey & Fischer, 2009). Fourth, we investigated whether acoustic divergence may just reflect genetic drift. In this scenario, we predict that acoustic distance between study sites is significantly correlated with genetic distance and that acoustic and molecular phylogenetic trees show a comparable branching pattern. Additionally, we investigated whether geographic distance can be used as proxy for genetic relationship.

## 2 MATERIALS AND METHODS

### 2.1 Study species, locations, trapping, and body measurements

**Figure 1:**
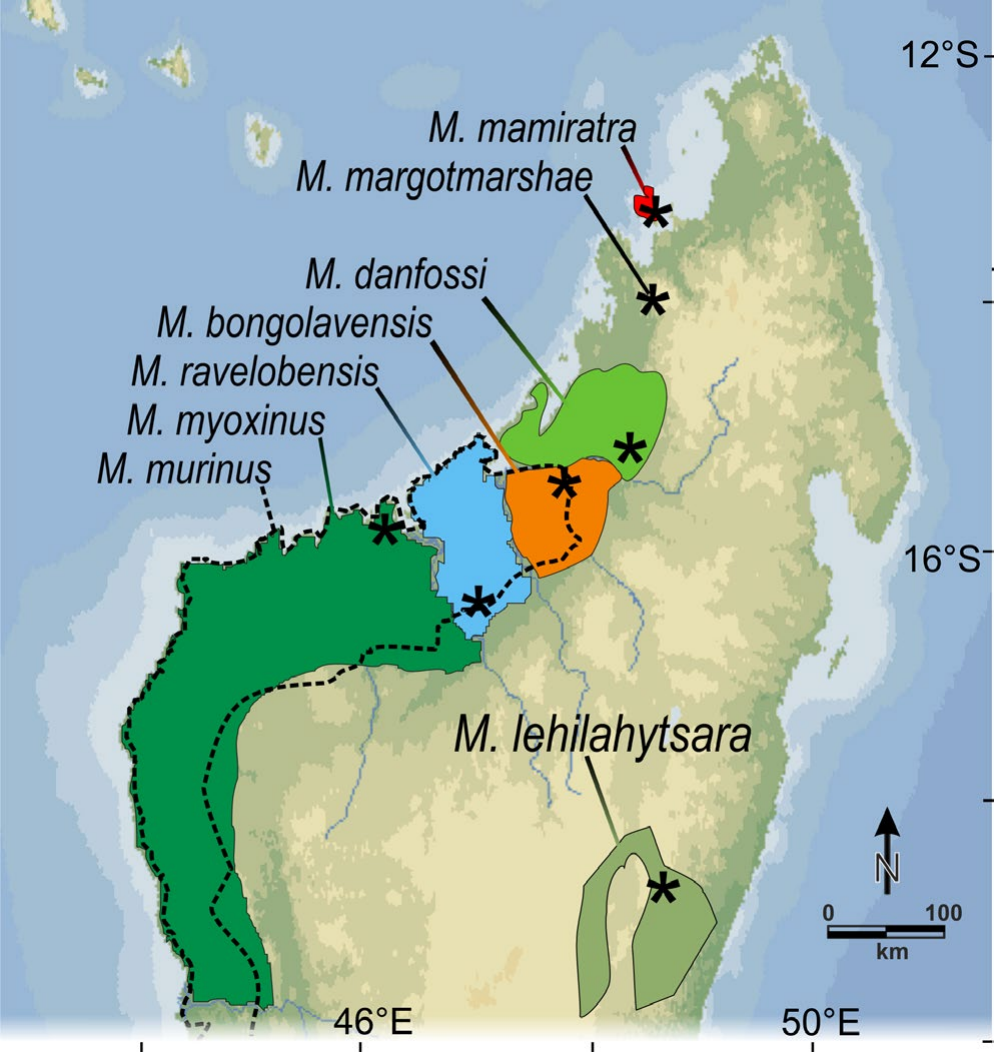
Distribution map of investigated species. The distribution range of *M. murinus* is indicated by the dotted line. Colored areas represent the distribution range for the other species. The distribution range of *M. margotmarshae* is not yet known. Asterisks represent sample locations

The study was conducted on six mouse lemur species at six different study sites in Madagascar from May to October 2015 and from June to October 2016 (Figure 1, Table 1). In addition, we included data from two further mouse lemur species. Data for *M. murinus* originated from Sharon Kessler (Kessler et al., 2014) and were recorded in Ankarafantsika National Park. Data for *M. lehilahytsara* were taken from the sound archive of the Institute of Zoology, University of Veterinary Medicine Hannover. Vocalizations for *M. lehilahytsara* were recorded from animals of the breeding colony at the animal facility at the Institute of Zoology, Hannover, Germany, that were descendents of founder animals originating from Andasibe.

**Table 1:** Locations of the eight mouse lemur species (*Microcebus* spec.), number of vocalizing dyads/subjects, and number of calls used in the acoustic analysis

Species	Location	No. of dyads/subjects	No. of calls	Audio recording
*M. mamiratra*	Ampasipohy, Lokobe National Park: 13°24′17.79″S, 48°20′37.11″E	10	93	SMX‐II weather‐proof microphones linked to Song Meter
*M. margotmarshae*	Ankaramibe forest: 13°58′30.91″S,48°10′39.03″E	11	157	
*M. danfossi*	Anjiamangirana: 15°10′01.20″S, 47°46′42.53″E	9	98	
*M. bongolavensis*	Marosely forest: 15°39′55.12″S, 47°34′40.08″E	11	157	
*M. ravelobensis*	Ankarafantsika National Park: 16°06′57.70″S, 47°05′49.82″E	11	95	
*M. myoxinus*	Bombetoka forest: 15°51′05.43″S, 46°15′37E	9	100	
*M. murinus*	Ankarafantsika National Park: 16°06′57.70″S, 47°05′49.82″E	12	157	D1000X Bat detector
*M. lehilahytsara*	Andasibe^a^: 18°54′00.37″S, 48, 26′55.26″E	5	57	bU30 Bat detector linked to a laptop equipped with a digital/analog converter card DAQ Card‐6062E

^a^ Note that the animals of *M. lehilahytsara* were recorded in the facility of the Institute of Zoology, but the founders of this colony originated from the location of Andasibe.

^b^ Sound recordings were taken from the sound archive of the Institute of Zoology.

The eight mouse lemur species live in different forest types. Whereas *M. murinus*, *M. danfossi, M. bongolavensis, M. ravelobensis*, and *M. myoxinus* live in deciduous dry forest, *M. margotmarshae*, *M. mamiratra,* and *M. lehilahytsara* live in low‐altitude or mid‐altitude evergreen humid forest (Du Puy & Moat, 1996). The study species belong to four phylogenetic clades (Louis & Lei, 2016; Figure 3): Clade 1 includes *M. murinus*; clade 2, *M. danfossi, M. bongolavensis,* and *M. ravelobensis*; clade 3, *M. margotmarshae* and *M. mamiratra*; and clade 4, *M. myoxinus* and *M. lehilahytsara.* Morphometric data were taken from body measurements of the captured wild study subjects except for eight *M. lehilahytsara* for which body measurements were available from the weekly health routines in the breeding colony of the animal facility of the Institute of Zoology. The following measurements related to body size and vocal tract morphology (Ey et al., 2007; Masters, 1991; Plotsky et al., 2013) were obtained: head length (from snout tip to occipital), head width (from the back of the basis of the left ear to that of the right ear), snout length (distance from the tip of the upper jaw to the anterior margin of the fleshy orbit), body size (distance from the neck to the basis of the tail), and the body mass of the individual.

### 2.2 Experimental setting and animals

For six of the eight studied species (*M. danfossi, M. bongolavensis, M. ravelobensis, M. margotmarshae, M. mamiratra, and M. myoxinus*), 12 dyads per species were observed. At each field site, 18 males and six female mouse lemurs were captured to form six male–male and six male–female dyads. Each mouse lemur was included in one dyad. Mouse lemurs were trapped using Sherman traps or caught by hand. Dyad partners were selected so that their body size matched and capture points were as far away as possible (median capture distance 244 m for mf‐dyads, 350 m for mm‐dyads) to minimize chances that dyads of familiar animals were put together. In each dyad, one animal was marked by a fur cut on its tail to be distinguishable. Dyad partners were housed together in a 1‐m^3^ cage that was placed on the forest ground in vicinity to the research camp. The cage was equipped with wooden bars and two sleeping sites. Water was provided ad libitum in a water bottle, and animals were fed with bananas at the beginning of each night. Arthropods were naturally available when they entered the cage. Observations were conducted between 6 p.m. and 9 p.m. for three consecutive nights (procedure matches to Hasiniaina et al., 2018). The observer sat 2–4 m in front of the cage wearing a dimmed headlamp while observing the animals. Behavior was recorded using the scan sampling method (15‐s scans) according to Altmann (1974). After the experiments, the mouse lemurs were released at the locations where they had been captured.

Vocalizations of *M. murinus* were recorded as playback stimuli for a study on kin recognition in female mouse lemurs (see Kessler et al., 2014). The animals were trapped with Sherman traps at the Ankarafantsika National Park and were temporarily kept in cages in the forest close to the research camp. *M. murinus* were housed either singly in cages (cage size: 0.5 m × 0.5 m × 1 m) connected by two passages to allow social encounters, or in small groups (cage size: 1 m × 0.5 m × 1.2 m) of up to four individuals. Food and water were provided as described above. The mouse lemurs were released at their capture locations after five nights on average. For the present study, we used recordings from 12 females uttered during social encounters.

Vocalizations of four captive male–female dyads and one male–male–female group of *M. lehilahytsara* were available from the sound archive of the Institute of Zoology. For call recordings, the animals were transferred from their home cage to a test cage in a sound‐attenuated room. The setup consisted of two cages, which were connected by a door. The sleeping boxes of the animals were fixed to the respective cage and opened. The calls were recorded during social interactions of the animals. The observer sat 1–2 m away from the test cage and observed the animals. An experimental session was conducted at the start of their activity phase and lasted approximately 60 min/day. Afterward, the animals were brought back to their home cages.

### 2.3 Audio recordings and acoustic analyses

Calls of *M. danfossi, M. bongolavensis, M. ravelobensis, M. margotmarshae, M. mamiratra,* and *M. myoxinus* were recorded using the same audio recording equipment, whereas the audio recordings for *M. murinus* and *M. lehilahytsara*, taken from the sound archive, were made with different microphones and recorders. For *M. danfossi, M. bongolavensis, M. ravelobensis, M. margotmarshae, M. mamiratra*, *and M. myoxinus*, vocalizations were recorded using two ultrasonic microphones (positioned at the cage walls; SMX‐II weather‐proof microphones, Concord, MA; frequency response of ± 5 dB from 15 to 40 kHz) connected to a Song Meter (Wildlife Acoustics, Model SM2+, Concord, MA; sampling rate of 192 kHz and 16‐bit resolution). For *M. murinus*, vocalizations were recorded using a D1000X Bat detector (positioned 2–4 m from the cage; frequency response of ± 3 dB from 5 to 40 kHz, sampling rate of 200 kHz, and 16‐bit resolution, Pettersson Elektronik, Uppsala, Sweden). For recording *M. lehilahytsara*, a U30 Bat detector (positioned at the cage wall; frequency response of ± 5 dB from 10 to 40 kHz; Schmidt, Hanke, & Pillat, 2000) was connected to a laptop equipped with a digital/analog converter card (DAQ Card‐6062E; sampling rate of 200 kHz and 12‐bit resolution). Although the three microphones differ somewhat in the lower frequency range for which a reasonably flat response characteristic is given, we expect only a minor effect on our data as the species (*M. murinus*) with the lowest fundamental frequency was also recorded with the microphone of best low‐frequency response. The somewhat different absolute sensitivities of the microphones were not relevant for our analysis since we did not analyze absolute amplitudes of animals moving freely inside the cages.

**Figure 2:**
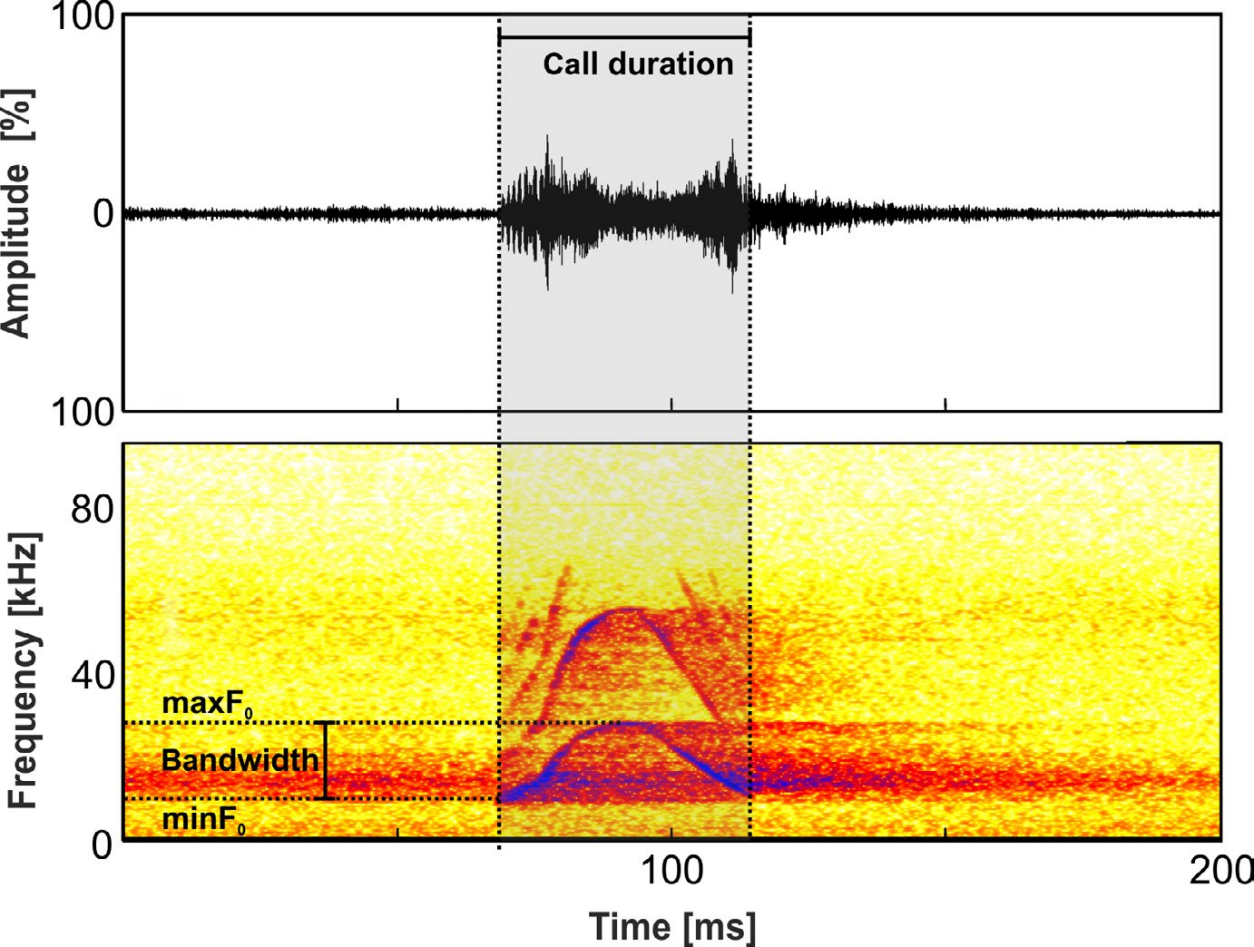
Sonogram and measured parameters of a Tsak call of *M. mamiratra*

Recorded files were audio‐screened with Audacity 2.2.2 and Batsound Pro 4.2. Across all studied species, the most common vocalization exchanged during agonistic conflicts was the so‐called Tsak call, recognizable by a uniform inverse U‐shaped frequency contour in the spectrograms (Hasiniaina et al., 2018; Zimmermann, 2010; Figure 2). Our analysis focused on this call type.

Except for *M. murinus,* calls were analyzed on dyadic level. This was necessary since mouse lemurs communicate in the high frequency to ultrasonic range, and it was impossible for the human ear to perceive the calls or to assign them reliably to an individual. In addition, the observations in dim light at night, and the fact that mouse lemurs have a facial open‐mouth display during agonistic interactions allowed no reliable assignment of calls to the respective individual by vision. In *M. murinus,* Tsak calls have a lower fundamental frequency, which enabled the observer to assign the calls to the respective individual. The number of Tsak calls emitted varied largely between the dyads. Although we tested 12 dyads for *M. danfossi, M. bongolavensis, M. ravelobensis, M. margotmarshae, M. mamiratra*, *and M. myoxinus*, not all dyads produced Tsak calls. To balance the data set, we selected a maximum of 15 Tsak calls per dyad or individual for further analysis. A total of 914 vocalizations from 78 dyads/subjects (5–12 dyads/subjects per species; distribution across species; see Table 1) of similar, and high, quality that were not overlapping with other sounds were analyzed using a custom‐built script in Praat (http://www.praat.org; Phonetic Sciences, University of Amsterdam, the Netherlands; Boersma, 2001). First, the audio recording was band‐pass‐filtered (filter frequency range: 75–60,000 Hz) and then time‐expanded by a factor of 10 using the “override sampling frequency” function of Praat to shift the ultrasonic vocalization into the human hearing range and to improve pitch tracking efficiency of the software. For each Tsak call, the following seven acoustic parameters were measured (Figure 2; Table S1): call duration (DUR), the percentage of the number of voiced frames (VOI), minimum (minF0), maximum (maxF0), bandwidth (BAND), mean (meanF0), standard deviation (sdF0), and mean slope (meanSLOPE) of fundamental frequency (F0; settings: “To pitch”; min pitch: 75 Hz; max pitch: 6,000 Hz; time steps: 0.01 s). Afterward, time expansion was reversed by multiplying all frequency values by 10 and dividing the temporal values by 10. For the meanSLOPE (Hz/s), the values were multiplied by 100. The raw data of the acoustic measurements are reported in Hasiniaina et al. (2020).

### 2.4 Uni‐ and multivariate statistical analyses

To describe the acoustic structure of the Tsak calls, we calculated the mean and the standard deviation for each measured parameter per dyad (dyad mean) and across all dyads per species (species mean). To investigate whether the parameters of the Tsak calls differed between the eight species, we first performed univariate ANOVAs with dyad/subject as random factor using the raw data set. To control for multiple testing, we performed the Fisher omnibus test (Haccou & Meelis, 1994). For pairwise comparison of the different species, we performed a post hoc test with Bonferroni correction.

To investigate to which extent the Tsak calls could be assigned to the respective species, we performed a stepwise discriminant function analysis. Since the discriminant function analysis required independent data, we used the dyad means for the analysis. We used the one‐leave‐out method for cross‐validation and the Kappa test to test the assignment of the classification with the original labels (Scheumann, Zimmermann, & Deichsel, 2007). The level of agreement is defined as follows: Cohen's kappa < 0.00 poor agreement; 0.00–0.20, slight agreement; 0.21–0.40, fair agreement; 0.41–0.60, moderate agreement; 0.61–0.80, substantial agreement; and 0.81–1.00, almost perfect agreement (Landis & Koch, 1977; Stemler, 2001). Additionally, we calculated a permutated discriminant function analysis, which allowed to control for dyad while using the raw data set (Mundry & Sommer, 2007).

To investigate whether species differ in morphometric parameters, a multivariate ANOVA was performed. To check whether acoustic differences between species may be explained by morphological differences, we conducted a Mantel test with 999 permutations correlating the acoustic Euclidean distance with the morphometric distance. To calculate the acoustic Euclidean distance and morphometric distance, we used the species means for each parameter. We standardized these means using a z‐transformation. Based on these standardized values, we calculated the Euclidean distance between the eight species for the acoustic and the morphometric data set, respectively. As Euclidean distance is measuring the dissimilarity between two species, large values reflect a greater dissimilarity between species.

To investigate the effect of forest type (dry vs. humid), we calculated linear mixed models for all parameters with forest type as predictor variable and dyad/subject nested in species as random factor using the raw data.

To investigate the relationship between the acoustic Euclidean distance, and genetic and geographic distance, we performed Mantel tests with 999 permutations. The genetic distance matrix across species was available from Olivieri et al. (2007) for seven of the species studied, namely *M. murinus, M. danfossi, M. bongolavensis, M. ravelobensis, M. lehilahytsara, M. mamiratra*, and *M. myoxinus*. Genetic distances between the seven species were expressed as the mean percentage of bp differences between individuals of different species. To calculate geographic distances across locations, GPS coordinates (longitude and latitude) were taken from the research camp at each field site using Garmin GPS MAP 60CVx. Based on these coordinates, geographic distances between all study sites were calculated in kilometers using GPS Visualizer (http://www.gpsvisualizer.com/calculators). In case of *M. lehilahytsara*, the coordinates of Andasibe (Table 1) were used from where the founder animals of the captive colony originated. Additionally, we calculated a partial Mantel test correlating acoustic Euclidean and genetic distance while controlling for geographic distance. We also correlated the genetic and geographic distance to check whether geographic distance can be used as proxy for genetic distance. To further compare molecular species divergence with acoustic species divergence, we build an acoustic tree using the acoustic Euclidean distance matrix, and compared it to a simplified cladogram derived from a previously published phylogenetic tree based on molecular data sets (Louis & Lei, 2016).

The software SPSS statistics 24.0 (IBM Corporation) was used to calculate the basic statistics, the uni‐ and multivariate ANOVA, and the stepwise discriminant function analysis. The Fisher omnibus test was calculated in Excel. To calculate linear mixed models, we used the software R (R version 3.1.1 (2014‐07‐10); R Core Team, 2014) with the packages “nlme.” The software PASSaGE (version v2; Rosenberg & Anderson, 2011) was used to calculate the Mantel tests. The acoustic tree was constructed using the software Neighbor of the PHYLIP package 3.69 (Felsenstein, 2012).

## 3 RESULTS

### 3.1 Variation in acoustic parameters between mouse lemur species

**Figure 3:**
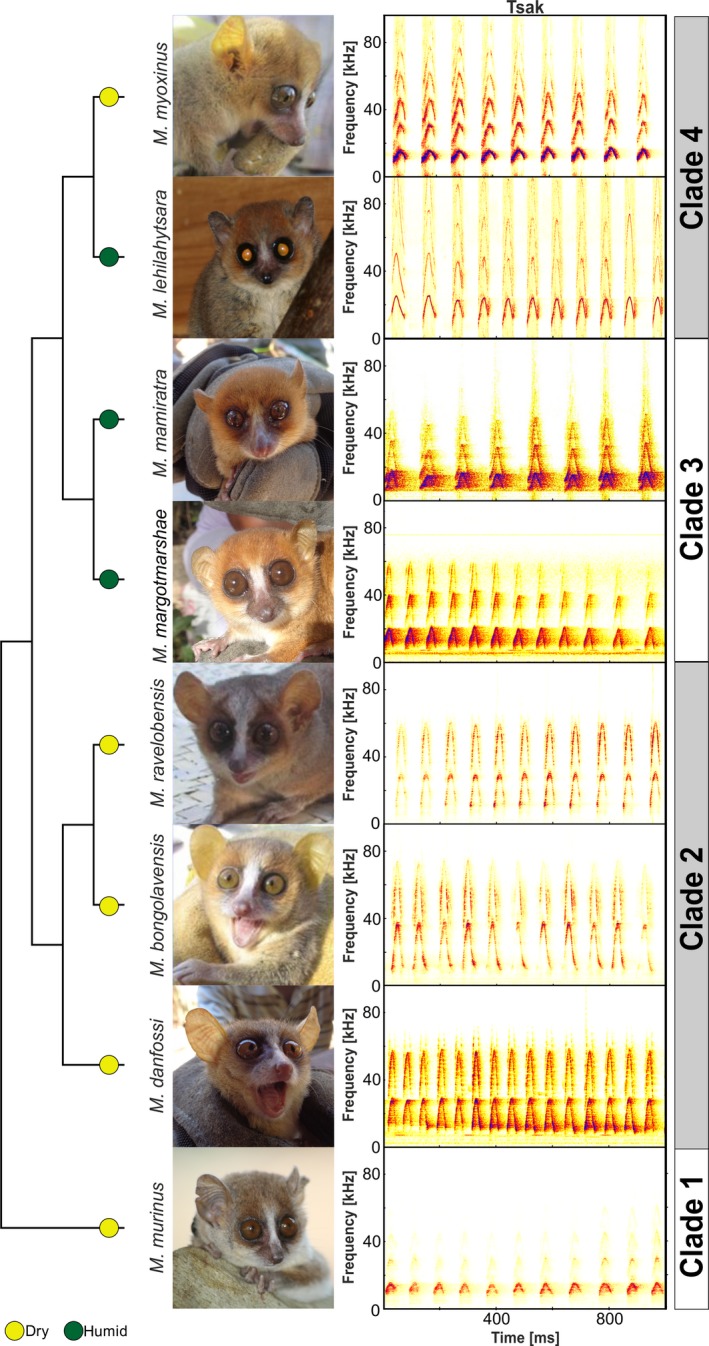
Diversity in Tsak calls of the eight studied mouse lemur species represented by photographs and spectrograms of the respective Tsak calls. The taxonomic cladogram is based on Louis and Lei (2016). Colored circle represents the forest type

The acoustic parameters of the Tsak calls differed between the eight mouse lemur species (*F* ≥ 2.931, *df* = 7, *N* = 78, *p* ≤ .009; Fisher's omnibus test: *F* = 471, *df* = 16, *p* < .001; Figure 3, Table 2). Post hoc tests revealed that call duration was longer in species of clade 1 and 2 (longest call duration in *M. murinus*: 38.2 ± 5.3 ms) compared with species of clades 3 and 4 (shortest call duration in *M. mamiratra*: 24.1 ± 5.0 ms; for statistics, see Table S2). In contrast, *M. murinus* showed significantly lower values of the minF0 (11.0 ± 1.0 kHz), maxF0 (16.2 ± 2.8 kHz), and meanF0 (13.7 ± 1.7 kHz) compared with almost all other mouse lemur species (Table S2). *M. lehilahytsara* showed the highest values of the minF0 (17.9 ± 3.4 kHz), whereas *M. bongolavensis* showed significantly higher values of maxF0 (31.1 ± 2.1 kHz) and meanF0 (25.0 ± 2.2 kHz) compared with almost all other mouse lemur species (Table S2). The sdF0, BAND, and meanSLOPE were significantly higher in *M. bongolavensis* (sdF0 = 5.0 ± 0.8 kHz, BAND = 15.2 ± 2.1 kHz, meanSLOPE = 891.8 ± 140.0 kHz/s) and *M. ravelobensis* (sdF0 = 4.7 ± 1.4 kHz, BAND = 13.7 ± 4.0 kHz, meanSLOPE = 850.2 ± 310.1 kHz/s) compared with the other mouse lemur species, while *M. murinus* showed the lowest value (sdF0 = 1.7 ± 0.7 kHz).

**Table 2:** Species means and standard deviations of the eight acoustic parameters measured in the eight studied species (N: number of dyads/subjects and *n* = number of calls)

Species	*M. murinus*		*M. danfossi*		*M. bongolavensis*		*M. ravelobensis*		*M. margotmarshae*		*M. mamiratra*		*M. lehilahytsara*		*M. myoxinus*	
	*N* = 12, *n* = 157		*N* = 9, *n* = 98		*N* = 11 *n* = 157		*N* = 11, *n* = 95		*N* = 11, *n* = 157		*N* = 10, *n* = 93		*N* = 5, *n* = 57		*N* = 9, *n* = 100	
	Mean	*SD*	Mean	*SD*	Mean	*SD*	Mean	*SD*	Mean	*SD*	Mean	*SD*	Mean	*SD*	Mean	*SD*
DUR (ms)	38.2	5.3	33.9	4.0	37.1	4.0	35.84	4.4	30.0	2.4	24.1	5.0	27.7	6.4	28.7	5.5
VOI (%)	99.6	5.3	94.2	5.3	94.8	5.3	93.8	5.3	95.0	5.3	95.5	5.3	100.0	5.3	99.1	5.3
minF0 (kHz)	11.0	1.0	14.0	0.8	15.9	2.1	11.9	1.8	14.0	1.3	15.1	2.6	17.9	3.4	16.9	2.1
maxF0 (kHz)	16.2	2.8	24.9	1.3	31.1	2.1	25.6	4.3	21.6	3.4	20.7	2.6	24.2	1.3	23.8	2.5
BAND (kHz)	5.2	2.0	10.8	1.5	15.2	2.1	13.7	4.0	7.6	2.4	5.7	1.5	6.3	2.4	6.9	1.6
meanF0 (kHz)	13.7	1.7	20.7	0.8	25.0	2.2	20.1	3.1	18.4	2.4	18.5	2.9	21.8	2.0	21.8	2.3
sdF0 (kHz)	1.7	0.7	3.4	0.4	5.0	0.8	4.7	1.4	2.5	0.9	1.9	0.8	2.0	0.7	2.2	0.6
meanSLOPE (kHz/s)	308.8	133.8	670.8	54.84	891.8	140.0	850.2	310.1	538.4	115.4	421.9	109.5	474.7	81.1	490.0	103.6

**Figure 4:**
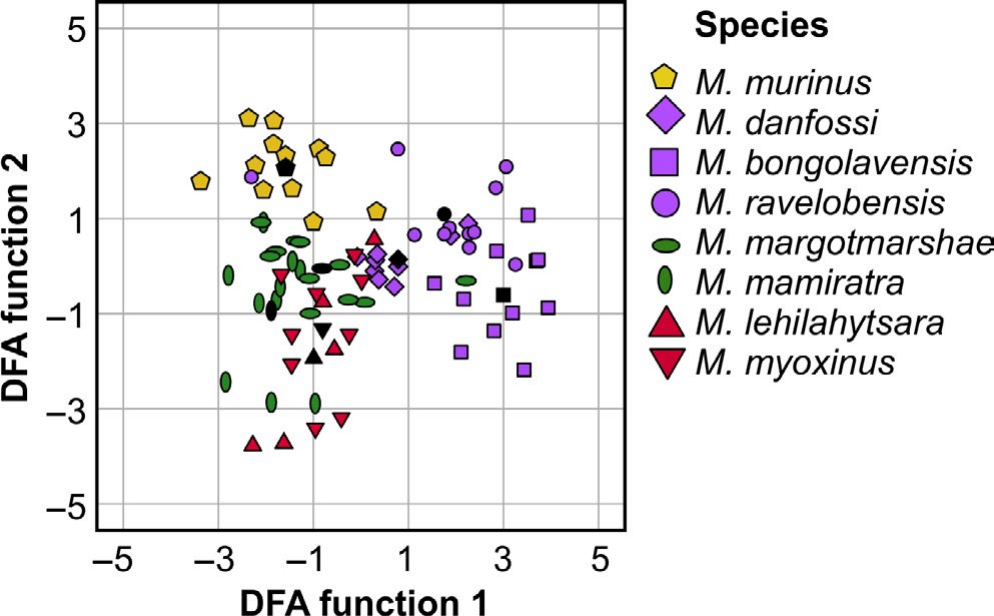
Scatterplot of the stepwise discriminant function analysis; different colors represent different clades. Black symbols represent the group centroid of the respective species

The stepwise discriminant function analysis based on dyad means selected BAND, maxF0, and DUR out of eight parameters to calculate three discriminant functions. Based on these functions, 67% of the Tsak calls were correctly classified to the respective species (cross‐validation: 60%; Figure 4). On species level, 73% of the calls for *M. ravelobensis,* 91% for *M. bongolavensis*, 83% for *M. murinus*, 82% for *M. margotmarshae,* 56% for *M. danfossi*, 50% for *M. mamiratra*, and 40% for *M. lehilahytsara* were correctly classified based on the original classification, which was above chance (binomial test: *p* ≤ .036; chance level: 6%–15%; see Table S3 for cross‐validated results). For *M. myoxinus*, we found a trend for correct classification (*p* = .075); thus, 33% of the calls were correctly classified. The Kappa test revealed a substantial agreement (0.616) between the original labels and the predictions of the DFA. Discriminant functions 1 and 2 correlated most strongly with frequency parameters characterizing the fundamental frequency (BAND, sdF0, maxF0, minF0, and meanF0 ≥ 0.700), whereas discriminant function 3 correlated most strongly with call duration (0.702) and meanSLOPE (−0.651). A pDFA based on the raw data set controlling for dyads/subjects supported these results. Significantly, more calls were correctly classified to the respective species than expected by chance (original classification: 54%, *p* = .001; cross‐validation: 43%, *p* = .001).

### 3.2 Effect of morphology on acoustic variation

Mouse lemur species differed in all morphometric measurements (multivariate ANOVA: *F* ≥ 7.3, *df* = 7, *p* ≤ .001; Table 3). As endpoints, *M. danfossi* and *M. mamiratra* were the heaviest species, whereas *M. myoxinus* was the lightest and smallest species. There was no significant correlation between acoustic Euclidean distance and the morphometric distance (Mantel test: *r* = .03, *p* = .897). Thus, morphometric similarity could not explain acoustic similarity.

**Table 3:** Species mean and standard deviation of the morphometric measurements of the eight studied species

Species		Head_length (mm)	Head_width (mm)	Body_size (mm)	Weight (g)	Snout_length (mm)
*M. murinus*	Mean	30.9	20.0	79.5	49.2	7.3
	*SD*	3.5	2.3	9.3	9.3	1.4
*M. danfossi*	Mean	36.9	21.7	77.2	65.4	7.4
	*SD*	1.4	0.9	2.6	6.8	0.6
*M. bongolavensis*	Mean	35.7	20.1	70.2	55.0	6.7
	*SD*	1.2	1.5	3.8	9.2	0.6
*M. ravelobensis*	Mean	35.5	21.0	74.7	58.6	6.4
	*SD*	1.4	1.6	7.1	7.9	1.1
*M. margotmarshae*	Mean	35.9	20.9	74.8	61.8	7.6
	*SD*	0.9	0.8	3.3	8.6	0.6
*M. mamiratra*	Mean	35.2	20.8	76.8	65.7	7.8
	*SD*	1.7	1.2	4.8	9.2	0.6
*M. lehilahytsara* ^a^	Mean	31.8	20.6	80.8	57.4	5.8
	*SD*	1.4	1.2	5.4	8.3	0.6
*M. myoxinus*	Mean	33.7	19.1	66.8	44.8	6.3
	sD	1.3	1.3	5.7	6.2	0.8

^a^ Note these body measurements were taken in captivity

### 3.3 Effect of forest type on acoustic variation

Results of the linear mixed models showed no significant effect of forest type on almost all acoustic parameters (*p* ≥ .291 for all parameters except DUR; Table S4). Call duration was significantly longer for species living in the dry versus the humid forest (*p* = .033). However, the Fisher omnibus test was not significant (*F* = 17.00, *df* = 16, *p* = .386). Thus, we found no clear evidence for an effect of forest type on Tsak structure.

### 3.4 Effect of genetic and geographic distance

**Figure 5:**
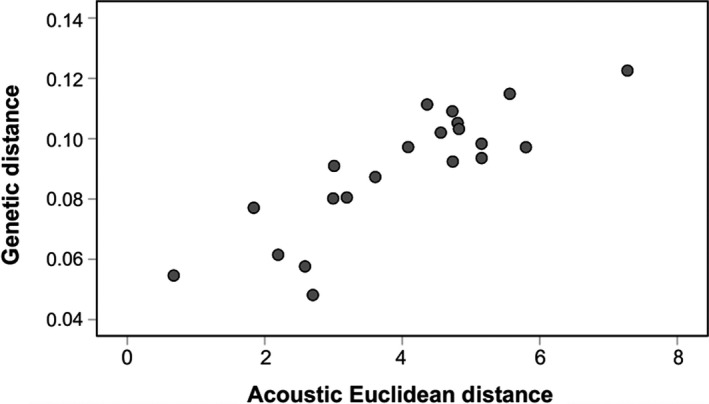
Relationship between genetic distances (mean proportion of bp differences between different species) and acoustic Euclidean distances for seven mouse lemur species. Circles represent species dyads

The results of the Mantel test showed a strong positive correlation between acoustic Euclidean distance and genetic distance (Mantel test: *r* = .854, *p* < .001; Figure 5). Thus, the smaller the genetic distance between species, the smaller the acoustic Euclidean distance between them, meaning that acoustic divergence is reasonably well predicted by genetic distance. This was also true when controlling for geographic distance (partial Mantel test: *r* = .844, *p* < .001). In contrast, no significant correlation was revealed between acoustic Euclidean distance and geographic distance (Mantel test: *r* = −.197, *p* = .448). Moreover, genetic distance and geographic distance were not correlated with each other (Mantel test: *r* = −.423, *p* = .123). 

**Figure 6:**
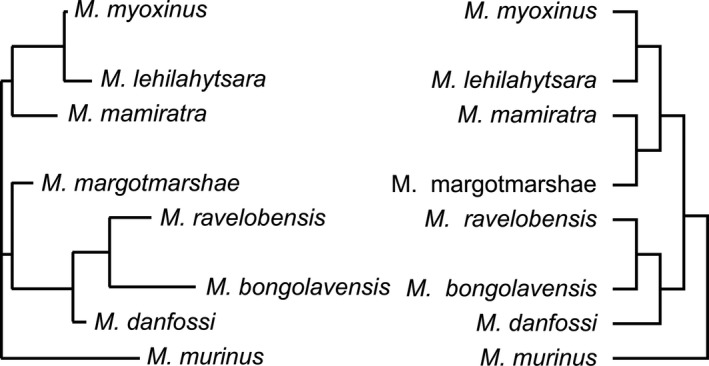
Comparison of the neighbor‐joining tree based on the acoustic Euclidean distance matrix (right) and a molecular tree (cladogram) of the model species based on Louis and Lei (2016) (left)

The acoustic and phylogenetic trees showed a high agreement in the branching pattern (Figure 6). In both trees, *M. murinus* stood alone. *M. ravelobensis, M. bongolavensis,* and *M. danfossi* formed a cluster. Within this, *M. ravelobensis* and *M. bongolavensis* were sister taxa. *M. myoxinus* and *M. lehilahytsara* also formed a cluster in both trees. The only differences occurred in the branching pattern of *M. mamiratra* and *M. margotmarshae*. Based on the acoustic tree, both species did not build a separate cluster, but *M. mamiratra* was paraphyletic associated with the cluster of *M. lehilahytsara* and *M. myoxinus.*

## 4 DISCUSSION

The eight mouse lemur species differed in the acoustic structure of their Tsak calls. This acoustic phenotypic variation could be explained by stochastic processes such as genetic drift, whereas morphometric differences between species or ecological selection did not account for the present findings. There was no correlation between the morphometric distance and the acoustic Euclidean distance of the tested species, nor did forest type predict the acoustic structure, or acoustic variability. In contrast, acoustic Euclidean distance correlated strongly with genetic distance and acoustic and molecular phylogenetic trees showed high agreement in their branching patterns. This indicates that genetic drift is a main driving factor for generating species‐specific call signatures in mouse lemur species.

Species‐specific signatures in social calls were found in various vertebrate species (e.g., Amézquita, Flechas, Lima, Gasser, & Hödl, 2011; Campbell et al., 2010; Irwin et al., 2008; McNett & Cocroft, 2008; Podos, 2010; Wilkins et al., 2013). However, the majority of studies focused on mating calls that can lead to, and maintain, reproductive isolation of species. *M. murinus*, *M. ravelobensis,* and *M. lehilahytsara* have been shown to differ in the acoustic structure of mating calls (Zimmermann, 2016; Zimmermann, Vorobieva, Wrogemann, & Hafen, 2000). Playback studies revealed that *M. ravelobensis* showed more attention to playbacks of conspecific or allopatric mating calls than to playbacks of the mating calls of the sympatric species (*M. murinus*; Braune et al., 2008) providing evidence for a perception of call divergence. However, the species‐specific signatures in Tsak calls disclosed in our study demonstrate that acoustic divergence is not restricted to mating calls only but is also present in agonistic calls. Thus, calls outside of the mating context may also be important for species recognition. This is consistent with studies on distress calls in wood mice (Ancillotto et al., 2017) or echolocation calls in some bat species (e.g., Bastian & Jacobs, 2015; Schuchmann & Siemers, 2010; Übernickel, Tschapka, & Kalko, 2013). Playback experiments in bats already showed that they can discriminate echolocation calls of their own species, or population, from those of other bat species, or populations (e.g., Bastian & Jacobs, 2015; Dorado‐Correa, Goerlitz, & Siemers, 2013; Schuchmann & Siemers, 2010; Übernickel et al., 2013). In *M. murinus*, playback experiments investigating auditory lateralization using Tsak calls of different mouse lemur species were performed (Scheumann & Zimmermann, 2008). In this study, mouse lemurs showed a lateralized response only to conspecific, but not to heterospecific, Tsak calls, suggesting that the species processed conspecific Tsak calls differently compared with heterospecific ones. Yet, further studies are needed to clarify to which extent the present species‐specific differences are discriminated.

Species‐specific differences in Tsak structure cannot be explained by differences in morphological traits related to the vocal tract, which is in accordance with studies in greenish warblers (Irwin et al., 2008). Likewise, forest type did not predict acoustic variation between species. We admit that in comparison with studies in amphibians or insects, our sample size with three dry forest and five humid forest species is a limited data set, and therefore, these negative results have to be interpreted cautiously. However, eight genetically distinct species are a large sample size for a primate study (for comparisons, see Adret et al., 2018; Hammerschmidt & Fischer 2019; Meyer et al., 2012; Thinh et al., 2011). Our result is in agreement with studies in mice and marmots who found no effect on habitat/climate on the acoustic structure of the calls (Campbell et al., 2010; Daniel & Blumstein, 1998) and with findings in warblers, which found also no correlation between acoustic data and habitat openness (Irwin et al., 2008). In contrast, effects of the environment have been reported from other mammalian and bird species (e.g., Baker, 2006; Berg, Brumfield, & Apanius, 2006; Boncoraglio & Saino, 2007; Ey & Fischer, 2009; Schneider et al., 2008; Sun et al., 2013). However, the meta‐analysis of Ey and Fischer (2009) showed that an influence of the environment on call structure was not widespread as expected and that the studies differed regarding general rules for call adaptation.

Our central finding that the evolution of species‐specific call differences in mouse lemurs is best explained by genetic drift is supported by the high positive correlation between acoustic and genetic distance even when controlling by geographic distance and by the high agreement in the branching patterns in the acoustic and molecular phylogenetic trees. This finding highlights that agonistic vocalizations do contain taxonomic and phylogenetic signatures (Doyle, 1978). This is in line with numerous studies in fish, insects, anurans (e.g., Amézquita et al., 2011), birds (e.g., Illera et al., 2018), and mammals (e.g., Campbell et al., 2010; Fischer & Hammerschmidt, 2020). On the other hand, we found no correlation with geographic distance, which is in contrast to studies on two singing mice species (Campbell et al., 2010), greenish warblers (Irwin et al., 2008), and crested gibbons (Thinh et al., 2011). In the latter studies, geographic distance was correlated with genetic distance. Results similar to our finding in mouse lemurs were found in other primate species (Meyer et al., 2012) and in Amazonian frogs (Amézquita et al., 2011) in which acoustic distance was strongly correlated with genetic distance but only weakly correlated with geographic distance. This shows that genetic distance was not mirroring geographic distance and thus the location of the study sites. Thus, the present data support a complex diversification pattern and demographic expansion history for the different clades within mouse lemurs. Indeed, different evolutionary scenarios are presently discussed for different mouse lemur lineages (Blair, Heckman, Russell, & Yoder, 2014; Hotaling et al., 2016; Louis & Lei, 2016; Olivieri et al., 2007; Schneider et al., 2010; Weisrock et al., 2010; Yoder et al., 2016). For *M. murinus* of clade 1, it is hypothesized that it originated from southwest Madagascar but expanded to the northwest following climatic and presumably vegetation changes in the late Pleistocene and early Holocene (Blair et al., 2014; Olivieri et al., 2007; Schneider et al., 2010). In contrast, it is hypothesized for the endemic forms that they evolved locally in the different humid forests of eastern, central, and northwestern Madagascar (Olivieri et al., 2007; Yoder et al., 2016). Correspondingly, local endemism within single Inter‐River‐Systems is discussed for the species of clades 2 and 3 (Olivieri et al., 2007; Weisrock et al., 2010). For the two species from clade 4 (*M. lehilahytsara* and *M. myoxinus*), a forest–grassland mosaic in the central highlands was suggested to form a transition zone and to act as major crossroad for ancestral lineages to move between the humid eastern (*M. lehilahytsara*) and the dry western forest habitats (*M. myoxinus*; Yoder et al., 2016).

Our comparative and integrative bioacoustics approach provides a framework for illuminating the role of vocalizations in cryptic species diversification and the evolution of primates. Vocalization can be used to clarify taxonomic or phylogenetic questions and also to monitor cryptic species for conservation. Our results match findings in singing mice (Campbell et al., 2010) and show that acoustic divergence is largely shaped by genetics. As a consequence, it can be hypothesized that species that split later in evolution must be more similar in call structure than those that split earlier. Further studies should test how this can be generalized to explain the speciation of cryptic mammals.

## CONFLICT OF INTEREST

We have no conflict of interest to declare.

## AUTHOR CONTRIBUTIONS

EZ initiated the study. EZ and SSch supervised the study. AFH and MRE collected the field data. SK contributed calls for *M. murinus*. UR contributed the genetic data. UR, SR, and BR organized data collection in the field. AFH performed the acoustic analysis. MS supervised the acoustic analysis. AFH and MS performed the statistical analysis. AFH, EZ, and MS wrote the draft of the manuscript. All authors revised the final manuscript.

## Supporting information

Table S1

Table S2‐S4

## Data Availability

The data set of the acoustic measurements can be uploaded on Dryad https://doi.org/10.5061/dryad.4tmpg4f65. The audio files are stored at the Institute of Zoology and are available on reasonable request.
