# Supplementary material for: Evolutionary significance of the variation in acoustic communication of a cryptic nocturnal primate radiation (Microcebus spp.)
Source: Ecol Evol. 2020 Mar 12;10(8):3784–97. doi: 10.1002/ece3.6177 (PMC7160168; doi:10.1002/ece3.6177)
Supplement: Supplementary file 1 — Table S1 [file ECE3-10-3784-s001.docx]

Table S1 Description of acoustic parameters. Frames = time windows in which the whole call was divided

| **Parameters** | **Unit** | **Definition** |
| --- | --- | --- |
| DUR | ms | Duration between the onset and the offset of a call. |
| VOI | % | Percentage of voiced frames of a call = number of frames where a fundamental frequency was detected divided by the total number of frames of the whole call. |
| minF0 | kHz | Minimum across all frames of the fundamental frequency of a call. |
| maxF0 | kHz | Maximum across all frames of the fundamental frequency of a call. |
| BAND | kHz | Difference between maxF0 and minF0 of a call. |
| meanF0 | kHz | Mean across all frames of the fundamental frequency of a call. |
| sdF0 | kHz | Standard deviation across all frames of the fundamental frequency of a call. |
| meanSLOPE | kHz | Mean inclination between two consecutive frames of the fundamental frequency of a call. |
